# Acetabular Distraction and the Cup-in-Cup Technique for the Management of Pelvic Discontinuity: A Case Report

**DOI:** 10.7759/cureus.99314

**Published:** 2025-12-15

**Authors:** Iturbide A Ponce de Leon Sandoval, Héctor A Soriano Solís, Alejandro Herce Santisteban, Jacobo Kerbel Sutton, Humberto González Ugalde

**Affiliations:** 1 Orthopedics and Traumatology Center, American British Cowdray (ABC) Medical Center, Mexico City, MEX; 2 Hip and Knee Replacement, American British Cowdray (ABC) Medical Center, Mexico City, MEX

**Keywords:** acetabular distraction, cup-in-cup technique, double-cup construct, paprosky iiib defect, periprosthetic pelvic discontinuity, revision total hip arthroplasty

## Abstract

Periprosthetic pelvic discontinuity is a rare but challenging complication in revision total hip arthroplasty (THA), particularly in the setting of severe acetabular bone loss. Restoration of the hip center and limb length, along with achieving implant stability, are critical for successful outcomes. We present a 68-year-old male with recurrent dislocations and a chronic flexible periprosthetic pelvic discontinuity of the left hip, likely originating from an intraoperative fracture during a prior THA revision, classified as Paprosky IIIB and American Academy of Orthopaedic Surgeons (AAOS) type IV. Imaging revealed a transverse fracture involving the iliopubic and ilioischial rami with significant bone loss. The patient underwent revision arthroplasty using acetabular distraction combined with a modular double-cup construct. A trabecular metal shell was used as a super-augment, and a smaller dual-mobility cup was cemented within the shell to restore the hip center, offset, and limb length. Postoperatively, the patient achieved stable fixation with restoration of limb length and hip biomechanics. Early functional outcomes were favorable, and radiographs demonstrated maintenance of the hip center without evidence of loosening. Acetabular distraction with a modular double-cup construct is an effective strategy for managing chronic flexible periprosthetic pelvic discontinuity with severe acetabular bone loss, providing immediate mechanical stability, restoring the hip center and offset, and offering a reproducible alternative to traditional reconstruction methods.

## Introduction

Total hip arthroplasty (THA) is a highly successful procedure that provides pain relief and functional improvement in patients with degenerative hip conditions. However, revision THA is expected to increase substantially, with a projected doubling of procedures in the United States by 2030 [1]. Revision surgery is particularly challenging in the presence of severe acetabular bone loss or complex pelvic defects, as achieving stable fixation and restoring hip biomechanics can be difficult [1,2].

Pelvic discontinuity (PD) refers to the loss of structural continuity between the superior and inferior portions of the pelvis. It may occur acutely during implant impaction or removal, or progressively due to osteolysis and implant loosening [1,2]. The remaining bone stock in such cases is frequently compromised, with variable biological healing potential and defect morphology, making stable fixation particularly challenging [3]. PD is commonly classified as type IV according to the American Academy of Orthopaedic Surgeons (AAOS) or as type IIIB according to Paprosky, often associated with substantial bone loss and medial migration of the acetabular component [1,4]. It can also be categorized as acute or chronic, and as stiff or flexible, depending on motion at the discontinuity site and the biological potential for healing [1].

Accurate preoperative diagnosis is critical, as unrecognized or iatrogenically created PD is a frequent cause of revision failure [1,2]. Standard imaging includes anteroposterior and lateral pelvic views, as well as Judet projections, where characteristic radiographic findings include a visible transverse fracture line, medial migration of the inferior hemipelvis, and asymmetry of the obturator ring [5]. Martin et al. proposed practical diagnostic criteria based on these features, demonstrating that when combining anteroposterior, lateral, and Judet views, PD can be accurately identified in nearly all cases preoperatively [5]. Computed tomography (CT) scans further assist in assessing bone loss, host bone quality, and fracture morphology, while three-dimensional reconstructions can facilitate surgical planning [1,2].

Various reconstructive strategies have been described for PD. Historically, reinforcement cages and rings provided structural support and a framework for cemented liners [1]. More recently, modular techniques using highly porous metal implants have gained popularity. In a systematic review of acetabular reconstructions for chronic PD, Malahias et al. found comparable mid-term survivorship among cup-cage, custom triflange, and highly porous cup constructs, with slightly higher success rates for the latter two techniques, supporting their use in cases with severe bone loss [6].

The cup-in-cup and cup-on-cup techniques involve cementing a smaller lateral cup into a larger medial shell, allowing restoration of the hip center of rotation (COR), horizontal and vertical offset, and improved initial stability [7,8]. Blumenfeld et al. reported mean improvements of 11 mm in horizontal offset and 22 mm in vertical offset in Paprosky IIIA and IIIB defects, without early mechanical failures [7]. Wu et al. demonstrated that the cup-on-cup technique similarly provides both medial and lateral support, enhancing initial stability and offset restoration [8].

Trabecular metal augments and double-cup constructs further improve fixation, particularly in severe Paprosky IIIA and IIIB defects, by increasing surface area for bone ingrowth and restoring the anatomical hip center. Webb et al. reported no cases of aseptic loosening at a mean follow-up of 2.36 years in 20 double-cup constructs, with significant improvement in Harris hip scores (preoperative 28.2 vs postoperative 68.7) [9]. Flecher et al. showed that cementless trabecular metal shells with modular augments achieved a mean postoperative leg-length discrepancy of 8.3 mm and a modified Postel-Merle d’Aubigne score of 10.6, without mechanical failures [2].

Acetabular distraction has been described as a technique for managing flexible PD. By expanding the defect rather than compressing it, this method allows secure fixation of a high-porosity acetabular shell with multiple screws, preserving the potential for biological healing [1,2]. The technique was originally described by Sporer et al., who achieved stable fixation through distraction across the discontinuity using an oversized porous tantalum cup, allowing elastic recoil and press-fit stabilization. Subsequent studies have confirmed its reproducibility and favorable outcomes. Sheth et al. reported 91% radiographic ingrowth and 83% survivorship at a mean five-year follow-up, while Bingham et al. demonstrated a 97% aseptic loosening-free survivorship at two years despite persistent discontinuity in more than half of cases [10-12]. Collectively, these findings suggest that distraction-based strategies can achieve satisfactory initial stability, restore the hip center, and improve functional outcomes in complex revision scenarios [4,9].

In this report, we present a case of periprosthetic PD treated with acetabular distraction and a cup-in-cup double-cup construct, highlighting the surgical approach, technical considerations, and early clinical outcomes.

## Case presentation

We present the case of a 68-year-old male patient with a history of left and right total hip arthroplasties performed 25 and 15 years prior to presentation, respectively. The patient experienced his first left hip dislocation roughly three years after the initial arthroplasty. Over the following 17 years, he sustained a total of 11 dislocations of the left hip, with the most recent episode occurring a few months before presentation.

After experiencing recurrent dislocations, the patient was assessed at an outside institution, where revision of the left THA was performed with placement of a Quattro-type dual-mobility prosthesis.

During routine postoperative follow-up, imaging revealed a transverse fracture line involving both the iliopubic and ilioischial rami. The patient underwent serial radiological evaluations over the following months. Subsequently, he sustained a low-energy fall impacting the left side of his body, after which he reported persistent pain in the left hip, subjective instability, and restricted range of motion. Despite these symptoms, he continued participating in physical therapy and routine activities. Seeking a second opinion, the patient presented for further evaluation and management.

On physical examination, shortening of the left lower limb was evident. The range of motion of the left hip was limited, with flexion of 90°, extension of 5°, abduction of 20°, and adduction of 10°. A positive left Trendelenburg sign was noted. The patient exhibited an antalgic gait favoring the left lower extremity and required a Canadian crutch for ambulation.

**Figure 1:**
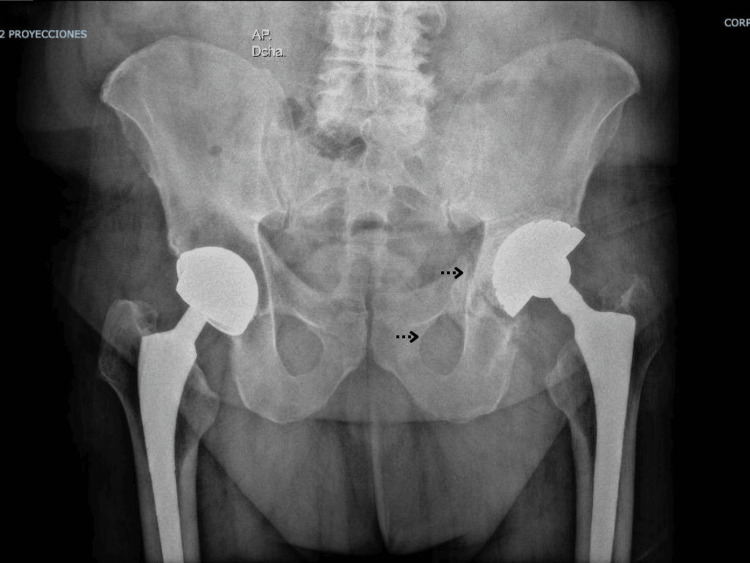
Preoperative AP pelvic radiograph showing pelvic discontinuity with medial migration of the inferior hemipelvis and asymmetry of the obturator ring.

**Figure 2:**
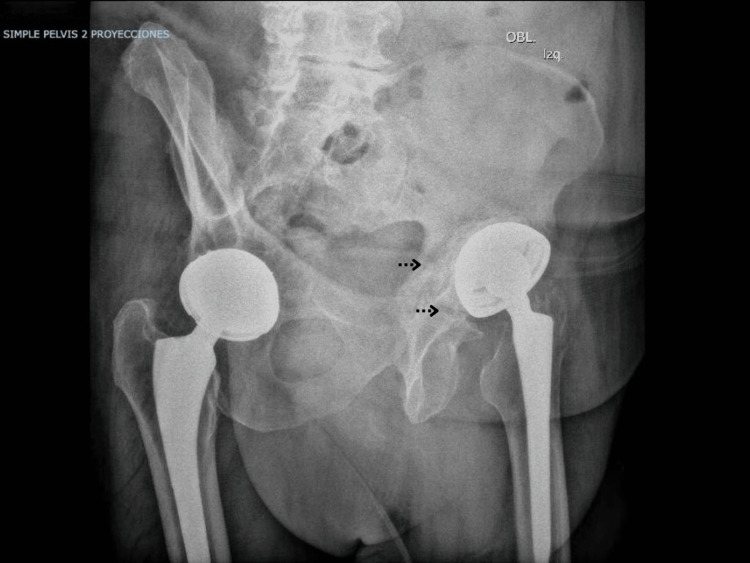
Judet oblique view demonstrating a transverse fracture line consistent with pelvic discontinuity and acetabular bone loss.

Pelvic anteroposterior (Figure 1) and left oblique (Figure 2) radiographs were obtained. A fracture line was identified in the left hemipelvis involving both the iliopubic and ilioischial rami. The left acetabular cup was cemented, with no signs of loosening.

**Figure 3:**
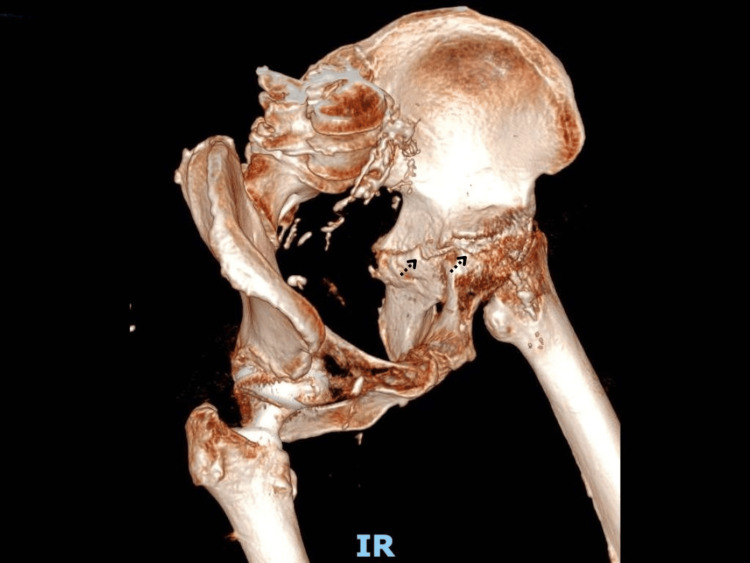
Preoperative CT with 3D reconstruction illustrating acetabular bone loss and discontinuity between the ilium and ischium.

Subsequent computed tomography (CT) of the pelvis confirmed a fracture line extending transversely through the acetabulum and involving both the iliopubic and ilioischial rami. Bone loss around the left acetabulum was also noted, likely related to the prior revision hip arthroplasty procedure. No signs of loosening of the acetabular component were evident on CT imaging. Three-dimensional CT reconstruction (Figure 3) further delineated the morphology, orientation, and extent of the fracture, providing critical information for preoperative planning and surgical decision-making.

**Figure 4:**
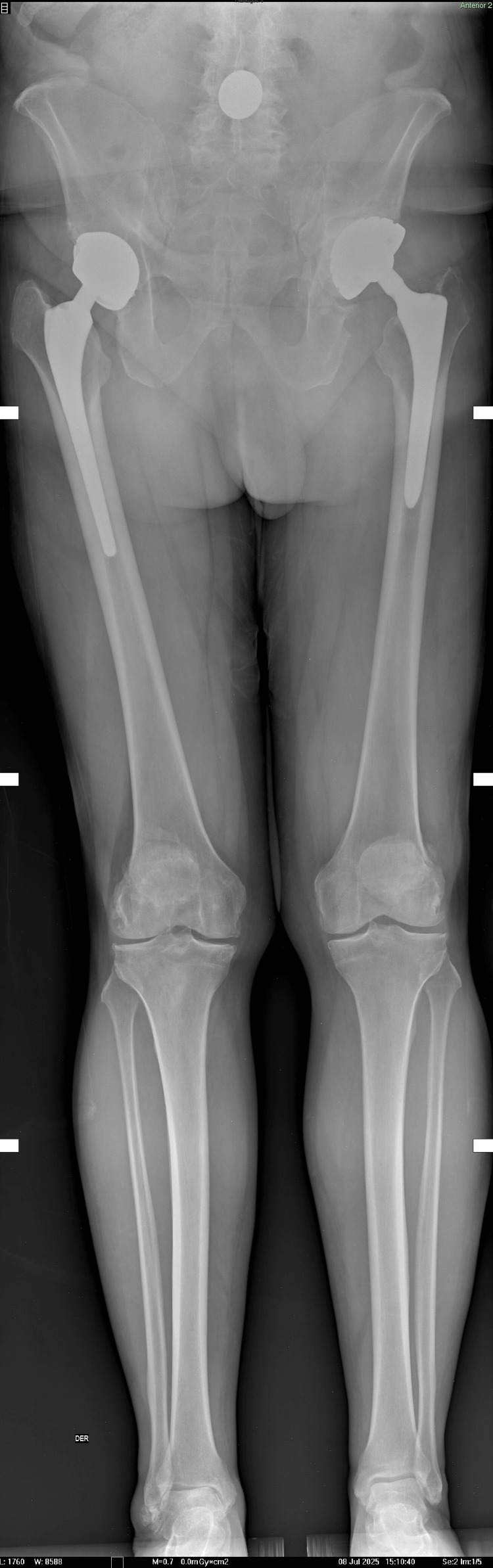
Mechanical axis assessment revealing left pelvic tilt and associated limb length discrepancy.

Mechanical axis evaluation (Figure 4) was used to further assess pelvic alignment and lower limb length, revealing a left pelvic tilt consistent with the patient’s clinical presentation and an associated limb length discrepancy. These findings were considered during surgical planning to optimize hip center restoration and achieve balanced alignment.

A diagnosis of periprosthetic PD of the left hip was established, classified according to the AAOS as type IV and the Paprosky classification as IIIB. Based on the fracture morphology and features of the PD, loosening of the acetabular component was presumed, despite the absence of clear radiologic evidence of loosening. This discontinuity was classified as a chronic flexible PD, likely originating from a fracture that occurred during placement of the acetabular component in the prior revision surgery and that failed to achieve osseous union.

Revision of the left THA was planned. Before initiating the procedure, a joint aspiration was performed under sterile conditions, yielding synovial fluid with normal macroscopic characteristics. The sample was collected and sent for microbiologic analysis and culture. The surgical approach was made through the previous lateral incision. During exposure, additional samples of periprosthetic soft tissue were obtained and sent for culture. Intraoperative assessment revealed adequate stability of the femoral stem and marked instability of the acetabular component. The dual-mobility femoral head was removed.

The acetabular cup was extracted using a cup extractor, with the goal of minimizing further bone loss. An osteotome was used to remove the remaining bone cement. Soft tissue and debris were cleared to fully expose the bone defect, confirming the diagnosis and identifying the PD as flexible based on the amount of motion observed between components. Sequential acetabular reaming was performed up to size 60, achieving adequate engagement with the subchondral bone. A size 66 trial component was inserted, demonstrating satisfactory stability.

An acetabular distraction technique was then applied to facilitate implantation of the definitive 66-mm Zimmer Biomet Trabecular Metal Acetabular Revision System (TMARS) cup. A set of self-tapping bone screws was placed in strategic orientations to achieve solid fixation of the acetabular component, with screw trajectories directed primarily toward the superior ilium for cortical purchase and toward the posterior ischium for additional stability.

A double-cup construct was selected to restore the hip COR and femoral offset and correct the limb length discrepancy. Trialing was performed with a dual-mobility cup placed inside the TMARS shell. After confirming the stability of the trial components, a cemented Quattro dual-mobility cup was implanted into the TMARS shell using the cup-in-cup technique, with appropriate anteversion and inclination. Stability tests, including flexion, extension, internal and external rotation, and pivoting maneuvers, demonstrated adequate fixation of the construct. The final implants included a 52-mm Quattro dual-mobility liner and a 28-mm +7 taper 12/14 stainless steel femoral head (Groupe Lépine).

The decision to use a dual-mobility cup of the same design as that implanted in the previous revision was based on the stable fixation of the femoral stem, which did not require revision. Replacing the stem would have required changing the femoral head and, consequently, selecting a different acetabular liner. By maintaining the existing femoral stem and head design, it was possible to implant a new dual-mobility cup of the same model, ensuring compatibility while avoiding unnecessary stem revision.

**Figure 5:**
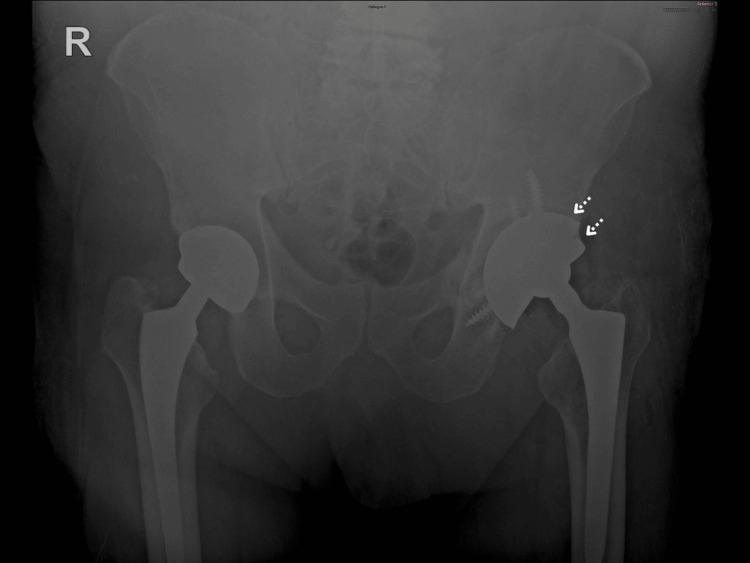
Immediate postoperative AP pelvic radiograph showing correct positioning of the double-cup construct and restoration of hip biomechanics.

An immediate postoperative anteroposterior (AP) pelvic radiograph (Figure 5) demonstrates proper placement of the double-cup construct in the left hip. The acetabular component shows adequate fixation, with restoration of the hip COR, femoral offset, and correction of the limb length discrepancy. The dual-mobility cup demonstrates appropriate orientation in both anteversion and inclination.

**Figure 6:**
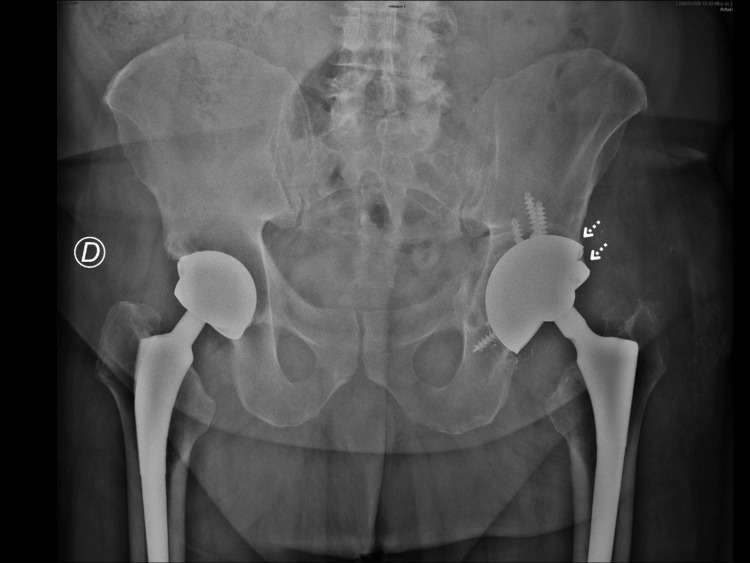
One-week postoperative AP pelvic radiograph showing stable alignment of the double-cup construct without evidence of loosening or malposition.

A follow-up AP pelvic radiograph (Figure 6) obtained one week later confirmed the maintained stability of the double-cup construct with no signs of loosening or malposition. All parameters, including implant positioning and alignment, remained satisfactory. Microbiological cultures from synovial fluid and periprosthetic tissue samples returned negative, effectively ruling out septic loosening.

## Discussion

Periprosthetic PD remains one of the most challenging scenarios in revision THA. In this case, the patient presented with a chronic flexible PD that likely originated from an acute intraoperative fracture during the prior revision THA, with significant acetabular bone loss. Successful management requires careful preoperative planning and a reconstructive strategy that provides immediate mechanical stability while allowing potential biological healing.

Historically, reinforcement cages and rings, such as the Burch-Schneider cage or Ganz ring, were commonly used to manage massive acetabular bone loss, providing structural support and a scaffold for cemented liners [1]. Despite their utility, these constructs often result in suboptimal restoration of hip biomechanics and a higher risk of mechanical complications. The development of highly porous metal implants, including trabecular metal shells, has improved osseointegration and long-term fixation in complex acetabular defects [2,4]. Malahias et al. reported that cup-cage constructs and custom triflange components achieved superior union and implant survival, 91.9% and 95.8%, respectively, highlighting the advantages of contemporary porous metal technology in achieving durable fixation and biological integration [6].

Modular techniques such as cup-in-cup and cup-on-cup have been increasingly applied to address severe medial and superior defects. The cup-in-cup approach involves impacting a medial porous shell into supportive host bone, followed by cementation of a smaller lateral cup to restore horizontal and vertical offset and the hip COR [7,8]. In the cup-on-cup technique, both medial and lateral cups contribute to initial stability and anchoring area, offering advantages in defects where conventional jumbo cups, augments, or cage constructs may not suffice [8].

The double-cup construct represents a further refinement in modular acetabular reconstruction. A larger super-augment supports the primary defect and maximizes the surface area for bone ingrowth, while a secondary modular shell is placed anatomically to restore the hip center [2,9]. Early reports demonstrate excellent survivorship from aseptic loosening and significant improvements in functional outcomes [2,9].

Acetabular distraction, as performed in this case, allows controlled expansion of the discontinuity rather than compression, facilitating secure fixation of the trabecular metal shell with multiple screws while preserving the potential for biological healing [1,2]. Recognizing a flexible discontinuity is critical to avoid overdistraction and ensure construct stability. Sporer et al. first described this technique using an oversized porous tantalum cup, with subsequent studies reporting high rates of radiographic ingrowth and implant survivorship [10-12].

Compared with traditional techniques, the combination of acetabular distraction and a modular double-cup construct achieved restoration of the hip center, limb length, and offset without requiring extensive structural grafting or custom triflange implants. This approach provides immediate mechanical stability, promotes osseointegration, and minimizes the morbidity associated with more invasive reconstructions [2,7,9].

## Conclusions

Chronic flexible periprosthetic PD represents a complex challenge in revision THA. In this case, the combination of acetabular distraction and a modular double-cup construct provided immediate mechanical stability, restoration of the hip center and limb length, and favorable early functional outcomes. This approach offers a reproducible and effective alternative to traditional reconstruction techniques, particularly in cases with severe acetabular bone loss and Paprosky IIIB defects. Further studies with larger cohorts and longer follow-up are warranted to confirm the durability and broader applicability of this technique.
